# Prognostic significance of annexin A2 and annexin A4 expression in patients with cervical cancer

**DOI:** 10.1186/s12885-016-2459-y

**Published:** 2016-07-11

**Authors:** Chel Hun Choi, Joon-Yong Chung, Eun Joo Chung, John D. Sears, Jeong-Won Lee, Duk-Soo Bae, Stephen M. Hewitt

**Affiliations:** Experimental Pathology Laboratory, Laboratory of Pathology, Center for Cancer Research, National Cancer Institute, National Institutes of Health, MSC 1500, Bethesda, MD 20892 USA; Department of Obstetrics and Gynecology, Samsung Medical Center, Sungkyunkwan University School of Medicine, 50 Irwon-dong, Gangnam-gu, Seoul, 135-710 Republic of Korea; Radiation Oncology Branch, Center for Cancer Research, National Cancer Institute, National Institute of Health, Bethesda, MD 20892 USA

**Keywords:** ANXA2, ANXA4, Prognosis, Survival, Uterine cervical neoplasms

## Abstract

**Background:**

The annexins (ANXs) have diverse roles in tumor development and progression, however, their clinical significance in cervical cancer has not been elucidated. The present study was to investigate the clinical significance of annexin A2 (ANXA2) and annexin A4 (ANXA4) expression in cervical cancer.

**Methods:**

ANXA2 and ANXA4 immunohistochemical staining were performed on a cervical cancer tissue microarray consisting of 46 normal cervical epithelium samples and 336 cervical cancer cases and compared the data with clinicopathological variables, including the survival of cervical cancer patients.

**Results:**

ANXA2 expression was lower in cancer tissue (*p =* 0.002), whereas ANXA4 staining increased significantly in cancer tissues (*p <* 0.001). ANXA2 expression was more prominent in squamous cell carcinoma (*p* < 0.001), whereas ANXA4 was more highly expressed in adeno/adenosquamous carcinoma (*p* < 0.001). ANXA2 overexpression was positively correlated with advanced cancer phenotypes, whereas ANXA4 expression was associated with resistance to radiation with or without chemotherapy (*p =* 0.029). Notably, high ANXA2 and ANXA4 expression was significantly associated with shorter disease-free survival (*p* = 0.004 and *p* = 0.033, respectively). Multivariate analysis indicated that ANXA2+ (HR = 2.72, *p* = 0.003) and ANXA2+/ANXA4+ (HR = 2.69, *p* = 0.039) are independent prognostic factors of disease-free survival in cervical cancer. Furthermore, a random survival forest model using combined ANXA2, ANXA4, and clinical variables resulted in improved predictive power (mean C-index, 0.76) compared to that of clinical-variable-only models (mean C-index, 0.70) (*p* = 0.006).

**Conclusions:**

These findings indicate that detecting ANXA2 and ANXA4 expression may aid the evaluation of cervical carcinoma prognosis.

**Electronic supplementary material:**

The online version of this article (doi:10.1186/s12885-016-2459-y) contains supplementary material, which is available to authorized users.

## Background

Cervical cancer is the third most common type of cancer in women worldwide and is the most prevalent female malignancy in many developing countries [1, 2]. Although vaccination and screening are excellent preventive options, the prognosis remains poor once the cancer has developed, particularly with bulky tumors or those with the adenocarcinoma cell type [3–5]. Clinical factors, such as stage, lymph node metastasis, and parametrial involvement, may serve as prognostic markers, but they are insufficient for accurately predicting survival. Thus, biomarkers, including molecular markers, are needed, and patient care would be improved considerably if tumor behavior could be prognosticated reliably at the time of initial diagnosis.

The annexins (ANXs) are a multigene family of calcium-regulated phospholipid-binding proteins [6] that share the ability to bind to negatively charged phospholipid membranes in a calcium-dependent manner. This binding is reversed by removing of calcium, and this reversible membrane-binding ability is thought to be important for vesicle aggregation and membrane organization [6, 7]. Twelve human ANX subfamilies (A1–A11 and A13) have been described, and each ANX has different calcium sensitivity and phospholipid specificity. In addition, the ANX are distributed differentially [7] and have various functions in cellular processes, such as calcium signaling, growth regulation, cytoskeletal organization, cell division, and apoptosis [6, 8]. Moreover, ANXs are involved in proliferation and invasion of tumor cells [9].

Up-regulation of annexin A2 (ANXA2) is associated with progression and metastasis of high-grade glioma [10] and hepatocellular [11], pancreatic [12], colorectal [13], lung [14, 15], and breast cancers [16], whereas down-regulation of ANXA2 occurs in patients with head and neck squamous cell carcinoma [17, 18], esophageal squamous cell carcinoma [19], and prostate cancer [20], indicating that ANXA2 may be a useful marker for the prognosis of these patients.

Annexin A4 (ANXA4), also called lipocortin IV and endonexin I, is associated with progression, invasion, migration, and drug resistance of cancers [21–23]. Prior studies have demonstrated that ANXA4 expression increases in colorectal cancer [22, 24], invasive renal clear cell carcinoma [25], and the clear cell carcinoma subtype of ovarian cancer [26]. In contrast, Xin et al. reported that ANXA4 expression decreases according to the progression of prostate cancer [27]. These data suggest that changes in ANXA2 and ANXA4 expression are associated with a particular tumor type, indicating that ANXs may be useful clinical biomarkers. However, knowledge on the clinical and prognostic significance of ANXA2 and ANXA4 expression in patients with cervical cancer is limited. In the present study, we investigated the prognostic significance of ANXA2 and ANXA4 in cervical cancers using immunohistochemistry and quantitative image analyses. Furthermore, we evaluated a predictive model of patient survival using combined ANX2 and ANX4 expression, as well as clinical variables.

## Methods

### Patients and tumor samples

We retrieved 336 patients with cervical cancer who were treated at the Department of Gynecologic Oncology, Samsung Medical Center, Sungkyunkwan University School of Medicine between 2002 and 2009. None of the patients had undergone previous treatment including radiation or chemotherapy. Patients with rare histology or an advanced stage treated primarily with radiation were excluded. As a control, 46 normal cervical epithelial samples were obtained from patients treated for benign uterine fibroids. The tissue specimens and medical records were obtained with informed consent of all patients and approval of the local research ethics committee (approval no. 2009-09-002-002 and 2015-07-122; Seoul, South Korea). Additional paraffin blocks were provided by the Korea Gynecologic Cancer Bank through Bio & Medical Technology Development Program of the Ministry of Education, Science and Technology, Korea (NRF-2012M3A9B8021800). This study was additionally approved by the Office of Human Subjects Research at the National Institutes of Health.

All patients were treated primarily by radical hysterectomy with or without pelvic/para-aortic lymph node dissection. Patients with risk factors, such as lymph node metastasis, parametrial involvement, positive resection margin, and stromal invasion of more than half of the cervix, received adjuvant radiotherapy with or without concurrent chemotherapy. Following treatment, the patients were followed up every 3 months for the first 2 years, every 6 months for the next 3 years, and every year thereafter. Disease-free survival (DFS) was assessed from the date of surgery to the date of recurrence or the date of the last follow-up. Overall survival (OS) was defined from the date of surgery to the time of death, or to the date of last contact.

### Western blotting

To detect the cellular localization of ANXA2 and ANXA4, CaSki and HeLa cells were subjected to fractionation, as described previously [28]. The cellular fractions (10 *μ*g) were separated by 4–12 % sodium dodecyl sulfate-polyacrylamide gel electrophoresis (SDS-PAGE) and transferred to a nitrocellulose membrane. After blocking for 1 h with 5 % nonfat milk in TBST (50 mM Tris, 150 mM NaCl, and 0.05 % Tween 20, pH 7.5), the membrane was probed with the following primary antibodies: anti-ANXA2 mouse monoclonal antibody (BD Biosciences, Oxford, UK; clone # 5/ANXAII, 1:3000 dilution) and anti-ANXA4 rabbit polyclonal antibody (Abcam, Cambridge, MA; cat. # ab33009, 1:1000 dilution). The membrane was incubated with the appropriate secondary antibodies for 1 h at room temperature. Immunoreactive bands were visualized using the SuperSignal Chemiluminescence kit (Thermo Scientific, Waltham, MA). Calnexin and lamin B1 were used as cytoplasm and nuclear extract indicators, respectively, as described previously [28].

### Tissue microarray and immunohistochemistry

Tissue microarrays (TMAs) were constructed from tissue blocks used for routine pathological evaluation. The original archived hematoxylin-eosin–stained slides were reviewed by a pathologist. Areas in each case with the most representative histology were selected, and a 0.6 mm tissue core was taken from each donor block and extruded into the recipient array. At least three samples from separate tissue blocks were taken from donor tissue blocks to fully represent each case. A section from each microarray was stained with hematoxylin and eosin and examined by light microscopy to check the adequacy of tissue sampling.

ANX immunohistochemical staining was performed using a standard streptavidin–peroxidase method, as described previously [29]. In brief, serial 4-*μ*m sections of the TMA were deparaffinized in xylene and rehydrated through a graded alcohol series. Heat-induced antigen retrieval was performed for 20 min in a pH 6.0 citrate antigen retrieval buffer (Dako, Carpinteria, CA) or in a pH 9.0 buffer for ANXA4 and ANXA2, respectively. Endogenous peroxidase activity was blocked with 3 % H_2_O_2_ for 10 min, and sections for ANXA4 were incubated with a protein block (Dako) for another 10 min. The sections were incubated with anti-ANXA2 mouse monoclonal antibody at a 1:5000 dilution for 30 min and with anti-ANXA4 rabbit polyclonal antibody at a 1:250 dilution for 2 h. The antigen-antibody reaction was detected with the Dako EnVision + Dual Link System-HRP (Dako) and DAB+ (3, 3′-diaminobenzidine; Dako). Tissue sections were lightly counterstained with hematoxylin and examined by light microscopy. Human renal tumors and human intestinal villi were taken as positive ANXA2 and ANXA4 controls, respectively. Negative controls were processed by omitting the primary antibody.

### Quantitative evaluation of immunostaining

Staining was quantitatively evaluated using computer-assisted image analyzing software (Visiopharm, Hoersholm, Denmark), as described previously [28]. In brief, slides were scanned using a whole slide scanner (NanoZoomer 2.0, Hamamatsu Photonics, Hamamatsu City, Japan) and imported into Visiopharm software using the TMA workflow. Staining intensity was categorized as 0, 1+, 2+, and 3+ according to the distribution pattern across cores. A brown staining intensity (0-negative, 1-weak, 2-moderate, and 3-strong) was obtained using a predefined algorithm and optimized settings. The overall immunohistochemical score (histoscore) was expressed as the percentage of positive cells multiplied by their staining intensity (possible range, 0–300). Quantitative digital image analysis was possible in all 366 cases with a wide range of histoscore. For the survival analysis, expression values were dichotomized (positive vs. negative) with the cut-off values showing the most discriminative power (histoscore of 94 for ANXA2 and 51 for ANXA4) (Additional file 1: Figure S1).

### In-silico analysis for GSE44001 and TCGA cervix

To examine the prognostic significance of the *ANXA2* and *ANXA4* mRNA expression, data from the Gene Expression Omnibus (GEO) and The Cancer Genome Atlas (TCGA) were analyzed, as described previously [29, 30]. A total of 300 patient samples were evaluable for GSE44001 (http://www.ncbi.nlm.nih.gov/geo/query/acc.cgi?acc= GSE44001), and 265 of the samples were also included in the immunohistochemical analysis of this study. The pan-cancer normalized form of the cervical cancer RNA‐seq data (version: 2015-02-24), which were obtained using Illumina HiSeq (Illumina, San Diego, CA, USA), were downloaded from TCGA Research Network for the TCGA data analysis (http://cancergenome.nih.gov/). mRNA expression values were dichotomized according to quartile values (lower than 25 percentile vs. higher than 75 percentile) for the survival analysis.

### Statistical analysis

The statistical analysis was performed using R software ver. 3.1.2. Student’s t-test or the Mann–Whitney *U*-test was used to compare the continuous variables between groups. Spearman’s rho coefficient analysis was used to assess correlations between parameters. Survival distributions were estimated using the Kaplan–Meier method and the relationships between survival and each parameter were analyzed with the log-rank test. A Cox proportional hazards model was created to identify independent predictors of survival.

To assess the predictive power of integrating the molecular data (ANXA2 and ANXA4) with clinical variables, we modified the random survival forest (RSF) method to include both clinical and molecular features [31]. We used clinical features (International Federation of Gynecology and Obstetrics (FIGO) stage, lymph node metastasis, lymphovascular invasion, stromal depth of invasion, parametrial involvement, and resection margin) to build the clinical RSF model. We then combined the molecular-level features with the clinical variables to build a new RSF model. We randomly split the samples into two groups for each set: 80 % as the training set and 20 % as the test set. The RSF models were built using the R package “Random Survival Forest” with the default parameters. The models were applied to obtain the test set for prediction, and the concordance index (C-index) was calculated using the R package “survcomp”. The C-index is a nonparametric measure to quantify the discriminatory power of a predictive model: a C-index of 1 indicates perfect prediction accuracy and a C-index of 0.5 is as good as a random guess [32]. The above procedure was repeated 100 times to generate 100 C-index values for each set. To compare performance between clinical variables only and the clinical variables plus the ANXA2/ANXA4 data, we used the Wilcoxon signed-rank test to calculate the *p* value. A *p* < 0.05 was considered significant.

## Results

### Clinicopathological patient characteristics

The clinicopathological characteristics of the 366 patients are presented in Table 1. Mean age of the patients was 48.9 ± 11.2 years. In total, 291 (86.6 %) patients were stage IIA or less and 45 (13.4 %) were stage IB2 or IIB. Tumor sizes ranged from 0.1 to 10.5 cm (mean, 3.21 cm). Postoperative radiotherapy with or without concurrent chemotherapy was administered to 160 patients (47.6 %). With a mean follow-up time of 66 months (range, 1–143 months), forty-six cases (13.7 %) developed recurrence and 20 patients (6.0 %) died.

**Table 1:** Correlation between annexin expression and the clinicopathological characteristics of patients with cervical cancer

Group	No	ANXA2		ANXA4	
		Mean histoscore [95 % CI]	*P* value	Mean histoscore (95 % CI)	*P* value
Normal	46	133 [111–156]	0.002	34 [21–46]	<0.001
Cancer	336	94 [88–101]		73 [65–81]	
Age					
<50 year	203	95 [87–103]	0.731	79 [68–89]	0.096
>50 year	133	93 [83–103]		65 [53–77]	
FIGO Stage					
≤ IIA	291	90 [83–97]	0.002	74 [66–83]	0.543
≥ IIB	45	121 [103–138]		67 [47–88]	
Cell type					
SCC	256	101 [93–108]	<0.001	51 [44–58]	<0.001
AD/ASC	80	73 [62–84]		145 [126–163]	
Tumor size					
≤4 cm	256	90 [82–97]	0.010	76 [66–85]	0.317
>4 cm	80	109 [96–121]		66 [49–83]	
LVSI					
Negative	202	88 [80–96]	0.019	83 [72–93]	0.005
Positive	133	104 [93–114]		60 [48–72]	
Depth of invasion					
<50 %	108	70 [60–80]	<0.001	70 [55–84]	0.565
≥50 %	228	106 [98–113]		75 [65–85]	
LN metastasis					
Negative	256	88 [81–95]	0.002	76 [66–85]	0.250
Positive	80	113 [100–127]		65 [50–81]	
PM involvement					
Negative	305	92 [85–98]	0.031	74 [65–82]	0.662
Positive	31	118 [95–141]		68 [42–94]	
Resection margin					
Negative	323	94 [87–100]	0.420	75 [66–83]	0.032
Positive	13	111 [66–157]		43 [15–71]	
Primary Treatment					
OP only	171	82 [73–90]	<0.001	78 [66–90]	0.354
OP + RT	70	110 [95–125]		71 [53–89]	
OP + CCRT	90	107 [95–119]		68 [54–82]	
Neoadjuvant	5	75 [45–105]		26 [8–59]	

*ANX* annexin, *FIGO* International Federation of Gynecology and Obstetrics, *SCC* squamous cell carcinoma, *AD* adenocarcinoma, *ASC* adenosquamous cell carcinoma, *LVSI* lymphovascular space invasion, *LN* lymph node, *PM* parametrium, *OP* operation, *RT* radiotherapy, *CCRT* concurrent chemoradiation

### ANXA2 and ANXA4 expression

To examine the prognostic significance of *ANXA2* and *ANXA4* mRNA expression, we analyzed the GEO and TCGA database. Patients with high *ANXA4* mRNA expression showed significantly poorer OS (*p* = 0.027) (Additional file 1: Figure S2).

**Fig. 1:**
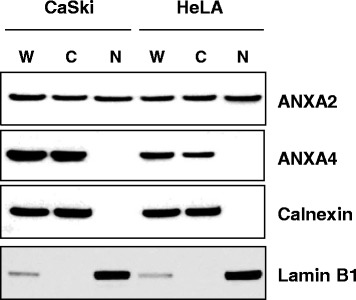
Subcellular localization of annexin A2 (ANXA2) and annexin A4 (ANXA4) in cervical cancer cell lines. Whole (W), cytosolic (C), and nuclear (N) fractions from CaSki and HeLa cells were analyzed by Western blot. Calnexin and lamin B1 were used as an index for the cytosolic and nuclear fractions, respectively

**Fig. 2:**
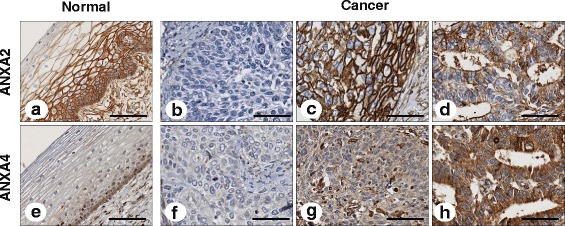
Representative immunohistochemical images of annexin A2 (ANXA2) and annexin A4 (ANXA4) in cervical cancer tissue. ANXA2 expression was strongly detected in the membranes of normal tissues (**a**), whereas ANXA4 staining was weakly observed in the cytoplasm of normal tissues (**e**). Negative staining demonstrated a lack of ANXA2 (**b**) and ANXA4 (**f**) expression. ANXA2 staining was mainly observed in the membranes of squamous cell carcinoma (**c**) and adenocarcinoma (**d**) cervical cancer tissues. ANXA4 staining was restricted to the cytoplasm of squamous cell carcinoma (**g**) and adenocarcinoma (**h**) (×200). Scale bar represents 50 *μ*m

Next, we performed Western blot using fractionated CaSki and HeLa cell lysates to examine the specificity anti-ANXA2 and anti-ANXA4 antibodies. ANXA4 was predominantly detected in the cytosolic fraction, whereas ANXA2 was detected in whole, cytosolic, and nuclear lysates (Fig. 1). The purities of the cytosolic and nuclear fractions were confirmed with calnexin and lamin B1, respectively. In addition, we performed immunohistochemistry in cervical cancer and normal tissues. ANXA2 staining was detected only in the membranes of normal cervical epithelium, whereas it was present in both the membranes and cytoplasm in cancerous tissues. ANXA4 staining was primarily observed in the cytoplasm. Representative examples of positive and negative staining are shown in Fig. 2. A significant increase in ANXA4 expression was detected in cancer tissues compared with that in normal cervix (mean histoscores; 73 vs. 34, *p* < 0.001). In contrast, ANXA2 expression was lower in cancer tissues than that in normal tissues (mean histoscores; 94 vs. 133, *p =* 0.002). A positive correlation was detected between mRNA and protein expression in patients with both protein and mRNA expression data from GSE44001 (Additional file 1: Figure S3).

ANXA2 and ANXA4 expression was associated with cell type. ANXA2 was more highly expressed in squamous cell carcinoma, whereas ANXA4 was expressed more prominently in adeno-/adenosquamous carcinoma (*p* < 0.001 and *p* < 0.001, respectively) (Table 1). The positive correlation between mRNA and protein expression was more prominent in squamous cell carcinoma for ANXA2 and in adenocarcinoma for ANXA4 (Additional file 1: Figure S3), suggesting that these ANXs potentially have different roles according to cervical cancer cell type.

**Fig. 3:**
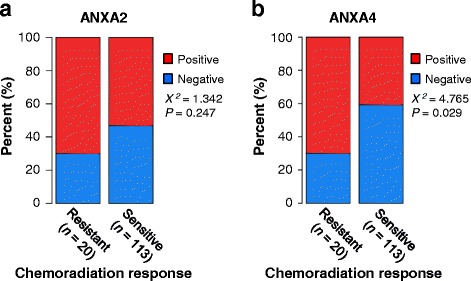
Association between chemoradiation response and annexin A2 (ANXA2) and annexin A4 (ANXA4) expression. ANXA4 expression was significantly correlated with resistance to chemoradiation (*p =* 0.029) (**b**), whereas ANXA2 expression was not (**a**)

ANXA2 expression was associated with a more aggressive cancer phenotype (Table 1). High ANXA2 expression was positively correlated with higher stage, large-sized tumors, lymphovascular space invasion, stromal invasion depth, lymph node metastasis, and parametrial involvement (*p* = 0.002, *p* = 0.01, *p* = 0.019, *p* < 0.001, *p* = 0.002, and *p* = 0.031, respectively). In contrast, ANXA4 expression was not associated with an aggressive phenotype, although it was more highly expressed in cancer tissue compared with normal tissue. To examine the association between ANX protein expression and chemo and/or radiotherapy resistance, we grouped patients receiving radiotherapy and/or chemotherapy into “resistant” (recurred within 3 years) or “sensitive” (no recurrence within 3 years) groups. As shown in Fig. 3, ANXA4 expression was significantly correlated with resistance to chemotherapy and/or radiation (*p* = 0.029).

### Prognostic significance of ANXA2 and ANXA4

**Fig. 4:**
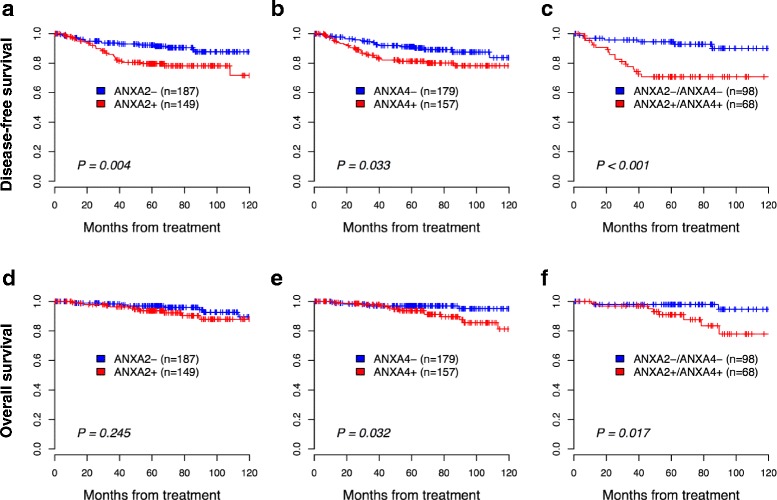
Kaplan-Meier plot for disease-free survival (DFS) and overall survival (OS) categorized based on annexin A2 (ANXA2) and annexin A4 (ANXA4) protein expression. High ANXA2 expression was associated with short DFS (*p* = 0.004) (**a**) but not with short OS (*p* = 0.245) (**d**). High ANXA4 expression was associated with short DFS (*p* = 0.033) (**b**) and OS (*p* = 0.032) (**e**). The association between high ANXA2/ANXA4 expression with DFS (**c**) and OS (**f**) was significantly different from that of low ANXA2/ANXA4 expression (*p* < 0.001 and *p* = 0.017, respectively). *P*-values were obtained from log-rank tests

The estimated five-year DFS and OS rates for the whole group were 87 % (95 % confidence interval [CI] 83–91) and 96 % (95 % CI, 93–98), respectively. ANXA2 and ANXA4 expression was significantly associated with poor DFS (*p* = 0.004 and *p* = 0.033, respectively) and OS (*p* = 0.245 and *p* = 0.032, respectively) (Fig. 4). The 5-year DFS rates were 80 and 81 % in patients with positive ANXA2 and ANXA4 expression, respectively, compared with 92 and 91 % in patients with negative expression. Similarly, 5-year OS rates were 94 and 94 % in patients with positive ANXA2 and ANXA4 expression respectively, compared with 97 and 97 % for patients with negative expressions*.* The combination of markers showed even greater discriminatory power and identified subgroups with 5-year DFS rates of 71 % vs. 95 % and 5-year OS rates of 91 % vs. 98 % using the ANXA2 and ANXA4 combination. The Cox proportional hazards model showed that expression of ANXA2 and combined ANXA2/ANXA4 expression remained an independent prognostic factor for DFS (hazard ratio [HR] = 2.72, 95 % CI, 1.41–5.27, *p* = 0.003; HR = 2.69, 95 % CI, 1.05–6.90, *p* = 0.039, respectively) (Table 2, Additional file 2: Table S1).

**Table 2:** Univariate and multivariate analyses of the association between prognostic variables and disease-free survival in patients with cervical cancer

Risk factor	Univariate		Multivariate	
	Hazard ratio [95 % CI]	*P* value	Hazard ratio [95 % CI]	*P* value
FIGO stage (> IIB)	2.44 [1.24–4.82]	0.010	1.38 [0.67–2.84]	0.381
Cell type (AD)	2.88 [1.62–5.14]	<0.001	4.58 [2.48–8.45]	<0.001
LN metastasis	4.13 [2.31–7.38]	<0.001	3.82 [2.03–7.18]	<0.001
Tumor size (>4 cm)	1.7 [0.92–3.15]	0.092	1 [0.51–1.95]	0.994
PM involvement	2.24 [1.05–4.81]	0.038	1.23 [0.55–2.76]	0.62
ANXA2+	2.33 [1.28–4.25]	0.006	2.72 [1.41–5.27]	0.003
ANXA4+	1.89 [1.04–3.41]	0.036	1.28 [0.64–2.56]	0.479
ANXA2+/ANXA4+	4.21 [1.75–10.09]	0.001	2.69 [1.05–6.90]	0.039

*CI* confidential interval, *ANX* annexin, *FIGO* International Federation of Gynecology and Obstetrics, *AD* adenocarcinoma, *LN* lymph node, *PM* parametrial

### Assessment of the prognostic value of ANXA2 and ANXA4

**Fig. 5:**
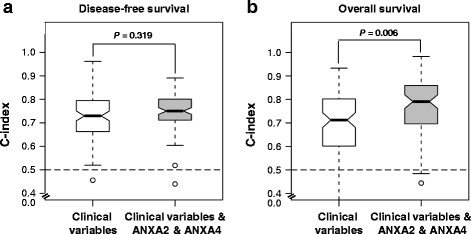
Comparison of survival predictive power of the clinical variables and the combined clinical and molecular data. We used the clinical variables (FIGO stage, lymph node metastasis, lymphovascular invasion, stromal depth of invasion, parametrial involvement, and resection margin) for the analysis. During 100 random splits, 80 % of all samples were used to train the model and the remaining 20 % were used as the test set for the C-index calculations. The white box highlights the model built from the clinical variables, and the grey-colored box highlights the models integrating the ANXA2 and ANXA4 data and clinical variables. The combined clinical and ANXA2/ANXA4 model predicting recurrence showed similar predictive power with the clinical variable model (**a**). However, the combined clinical/molecular-variable model showed better performance than that based only on clinical variables in the model predicting death (Mann-Whitney *U*-test, *p =* 0.006) (**b**). The dashed lines mark the C-index equivalent to a random guess (C-index = 0.5)

To examine whether molecular data and ANXA2 and ANXA4 protein expression provided additional prognostic power when used with the clinical variables, we compared the predictive models using only the clinical variables and the combined clinical/molecular variables. The combined clinical/molecular-variable model predicting death resulted in significantly improved power (mean C-index, 0.76; range, 0.73–0.79) compared to the clinical-variable-only model (mean C-index, 0.70; range, 0.68–0.73) (*p* = 0.006) (Fig. 5). For models predicting recurrence, the combined clinical and ANXA2/ANXA4 model showed similar predictive power (mean C-index, 0.76, 95 % CI, 0.75–0.78) to clinical-variable-only model (mean C-index, 0.75, 95 % CI, 0.73–0.77).

## Discussion

ANXA2 exists as a monomer or heterotetramer composed of two ANXA2 molecules and partner molecules and has four forms, including secrete, membrane-bound, cytoplasmic, and nuclear forms [33]. In the present study, levels of ANXA2 expression decreased in cervical cancer tissues compared to those in normal tissues; however, differences in subcellular localization were detected (Fig. 2). ANXA2 was detected very strongly near the cytosolic membrane in normal cervical epithelial specimens, whereas it was detected in both membranes and the cytoplasm in cervical cancer tissues. This finding suggests that ANXA2 may not just play a role as a Ca^2+^ binding protein on the membrane surface, but also as a component of dynamic trafficking pathways, such as exocytosis and endocytosis [6]. Enhanced trafficking pathways are an emerging feature of cancers during initiation and progression [34]. Concomitantly, the increase of ANXA2 expression was associated with a more aggressive phenotype among cervical cancer tissues examined in this study (Table 1). Nuclear ANXA2 was detected by Western blot in cervical cancer cell lines (Fig. 1). In general, ANXA2 expression in the nucleus is considered a cell-cycle-dependent phenomenon [33]. We also found that ANXA2 and ANXA4 were differentially expressed according to cell type, suggesting that each ANX potentially has a different role in squamous cell carcinoma or adenocarcinoma in patients with cervical cancer. Furthermore, we demonstrated that high ANXA2 and ANXA4 expression predicted poor survival in patients with cervical cancer, which was supported by the improved predictive power of a model using combined clinical-ANXA2/ANXA4-variables. These results suggest that ANXA2 and ANXA4 protein analyses can be a prerequisite in diagnoses of cervical cancers and may guide the patient therapy.

Few reports are available on ANX expression in cervical cancer or its correlation with prognosis. Jin et al. evaluated the significance of ANXA2 protein expression to predict the response to neoadjuvant chemotherapy in patients with cervical cancer [35]. ANXA2 protein expression correlates with tumor response to chemotherapy, and ANXA2 expression in stromal cells was an independent prognostic factor for DFS. No report has evaluated ANXA4 expression and prognosis of cervical cancer.

ANXA2 expression was associated with an aggressive phenotype, such as higher stage, large-sized tumors, lymphovascular invasion, invasion depth, lymph node metastasis, and parametrial involvement in the present study. ANXA2 promotes cell invasion in malignancies of the breast, brain, liver, and pancreas [10–12, 36, 37] and enhances cell motility and cell adhesion of prostate and hepatocellular carcinoma cells [38, 39]. One of the mechanisms enhancing cancer metastasis is the interaction between ANXA2 and its binding proteins, which play an important role in the tumor microenvironment [40]. ANXA2 binds with plasminogen and tissue plasminogen activator on the cell surface, leading to the conversion of plasminogen to plasmin, which plays a key role activating metalloproteinases and degrading the extracellular matrix components essential for metastatic progression. In addition, after binding to collagen I, cathepsin B and tenascin-C, ANXA2 assists in maintaining plasticity and rearrangement of the actin cytoskeleton which is important in metastasis [41, 42]. In support of these findings, ANXA2 siRNA or neutralizing antibodies significantly inhibit motility and invasion of ovarian cancer cells in vitro and in vivo [40].

Persistent infection of human papillomavirus (HPV) is highly associated with cancers arising in squamous epithelium, and approximately 90 % of cervical cancer cases are associated with HPV as a causative agent [43]. HPV must gain entry into host basal cells of the epithelium to deliver its double-stranded DNA to the nucleus and the HPV capsid proteins play a vital role in these steps [44]. However, the mechanisms of entry and the specific receptors directly involved in the internalization of oncogenic HPVs remains unclear. Dziduszko and Ozbun have shown that HPV16 particles interact with ANXA2 in association with S100A10 as a heterotetramer at the cell surface in a Ca^2+^-dependent and likely a heparin-sulfonated proteoglycan-dependent manner [45]. They confirmed the role of ANXA2 in HPV16 infection by showing that (i) early HPV16 binding results in extracellular translocation of ANXA2, (ii) ANXA2 cointernalizes and mediates intracellular trafficking of HPV16 and (iii) anti-A2 and anti-S100A10 antibodies block HPV16 infection at different stages of HPV16 infection. Notably, Woodham et al. have reported that small molecular inhibitors of the ANXA2 heterotetramer prevent HPV16 pseudovirions infection in HeLa cells [46]. These data indicate that ANXA2 may play a critical role in tumorigenesis, which could potentially be applied as a therapeutic target of cervical cancer.

The role of individual ANXs has been reported in various cancer types in previous studies. Mussunoor and Murray overviewed the role of each ANX on a variety of cellular functions including cell proliferation, apoptosis, angiogenesis, invasion and differentiation [9]. Except ANXA9 and ANXA13, changes in the expression of individual ANXs were observed in a diversity of cancers, including gastric carcinoma, colorectal cancer, pancreatic cancer, breast cancer, glioma, kidney cancer, hepatocellular, carcinoma, melanoma, but not in cervical cancer [9]. In order to assume the influence of HPV on ANX expression in cervical cancer, we analyzed the TCGA pan-cancer data. The level of *ANXA2* mRNA expression is relatively high in the cervical and head/neck cancers which are largely correlated with HPV infection, whereas *ANXA4* mRNA expression showed intermediate level in the both cancers (Additional file 1: Figure S4).

The association between ANXA2 and ANXA4 expression and specific HPV type infection could not be assessed in the present study due to the lack of clinical information about the specific HPV type infection in patients. Therefore, we analyzed the TCGA database in order to assess the relationship between *ANXA2* and *ANXA4* mRNA expression and specific HPV type infection. The HPV type information is only available from 22 patients with cervical cancer. *ANXA2* and *ANXA4* mRNA expression showed no difference among the HPV types (Additional file 1: Figure S5). Studies evaluating the relation between a different type of HPV and ANX function will give further insight about the role of ANXs in cervical carcinogenesis.

The poor survival in patients who expressed ANXA2 and ANXA4 seen in this study may have been due to the correlation with aggressive phenotype, particularly in those who expressed ANXA2. However, another important possibility is an association with radio/chemo resistance. ANXA4 expression was significantly associated with chemo/radio resistance in this study. Although the functions of ANXA4 are not completely known, many studies have identified the involvement of ANX in membrane permeability [47], exocytosis [48, 49], and regulation of ion channels [50]. These functions may explain the involvement of ANXA4 in modulating drug resistance through efflux of intracellular chemotherapy drugs in cancer cells. In addition to increased efflux, modulation of the transcriptional activity of nuclear factor kappa-light-chain-enhancer of activated B cells (NF-kB) has been suggested as a mechanism of chemoresistance. A previous study identified an association between ANXA4 and NF-kB transcriptional activity. Jeon et al. [51] showed that ANXA4 suppresses NF-kB transcriptional activity, which is significantly upregulated immediately after etoposide treatment. ANXA4 translocate to the nucleus together with p50 and imparts greater resistance to apoptosis stimulation by etoposide. They concluded that ANXA4 differentially modulates the NF-kB signaling pathway, depending on the interaction with p50 and intracellular Ca2^+^ levels. We also shown previously that overexpression and nuclear localization of ANXA4 are related to chemoresistance and poor survival in patients with serous papillary ovarian carcinomas [52]. In contrast to ANXA4, the association between ANXA2 and chemoresistance has not been well studied and remains unclear.

## Conclusions

In conclusion, our results show that ANXA2 and ANXA4 protein expression, alone or in combination, are independently poor prognostic factors of survival in patients with cervical cancer. This information can be helpful in the management of patients with cervical cancer. Patients who express high levels of ANXA2 and ANXA4 should be considered for closer follow-up or intensified adjuvant treatment.

## Abbreviations

AD, adenocarcinoma; ANXA2, annexin A2; ANXA4, annexin A4; ANXs, annexins; ASC, adenosquamous cell carcinoma; CCRT, concurrent chemoradiation; CI, confidence interval; DFS, disease-free survival; FIGO, International Federation of Gynecology and Obstetrics; GEO, Gene Expression Omnibus; HPV, human papillomavirus; HR, hazard ratio; LN, lymph node; LVSI, lymphovascular space invasion; OP, operation; OS, overall survival; PM, parametrium; RFS, random survival forest; RT, radiotherapy; SCC, squamous cell carcinoma; TCGA, The Cancer Genome Atlas; TMA, tissue microarray.

## Additional files

Additional file 1: Figure S1. Histoscore distribution of annexin A2 (ANXA2) and annexin A4 (ANXA4) expression using a quantitative image analysis. Dashed vertical lines indicate the chosen cut-off values (ANXA2 = 94 and ANXA4 = 51). Figure S2. Kaplan-Meier survival curves according to annexin A2 (ANXA2) and annexin A4 (ANXA4) mRNA expression. Data were retrieved from the GEO (http://www.ncbi.nlm.nih.gov/geo/query/acc.cgi?acc=GSE44001) and TCGA (RNA-seq databases (version: 2015-02-24). The mRNA expression values were dichotomized according to quartile values (lower than 25 percentile vs. higher than 75 percentile). Figure S3. Correlations between annexin A2 (ANXA2) and annexin A4 (ANXA4) mRNA and protein expression. Positive correlations were noted in both ANXA2 (*Spearman’s rho* = 0.273, *p* < 0.001) and ANXA4 (*Spearman’s rho* = 0.293, *p* < 0.001). A subgroup analysis showed a positive correlation between ANXA2 expression and squamous cell carcinoma (*Spearman’s rho* = 0.255, *p* < 0.001) and ANXA4 expression and adenocarcinoma (*Spearman’s rho* = 0.389, *p* = 0.002). Figure S4. *ANXA2* and *ANXA4* mRNA expression according to cancer types. (Data from cBioPortal Cancer Genomics: www.cbioportal.org). Figure S5. *ANXA2* and *ANXA4* mRNA expression according to HPV type infected. Data was retrieved from TCGA (RNA-seq database version 2015-02-24). (PPTX 1250 kb) Additional file 2: Table S1. Univariate and multivariate analyses of the association between prognostic variables and overall survival in patients with cervical cancer. (DOCX 15 kb)

## References

[1] Suh DH, Kim JW, Aziz MF, Devi UK, Ngan HY, Nam JH (2010). Asian society of gynecologic oncology workshop 2010. J Gynecol Oncol.

[2] Jemal A, Bray F, Center MM, Ferlay J, Ward E, Forman D (2011). Global cancer statistics. CA Cancer J Clin.

[3] Thomas GM (1999). Improved treatment for cervical cancer--concurrent chemotherapy and radiotherapy. N Engl J Med.

[4] Gien LT, Beauchemin MC, Thomas G (2010). Adenocarcinoma: a unique cervical cancer. Gynecol Oncol.

[5] Lee YY, Choi CH, Kim TJ, Lee JW, Kim BG, Lee JH (2011). A comparison of pure adenocarcinoma and squamous cell carcinoma of the cervix after radical hysterectomy in stage IB-IIA. Gynecol Oncol.

[6] Gerke V, Creutz CE, Moss SE (2005). Annexins: linking Ca2+ signalling to membrane dynamics. Nat Rev Mol Cell Biol.

[7] Liemann S, Huber R (1997). Three-dimensional structure of annexins. Cell Mol Life Sci.

[8] Gerke V, Moss SE (2002). Annexins: from structure to function. Physiol Rev.

[9] Mussunoor S, Murray GI (2008). The role of annexins in tumour development and progression. J Pathol.

[10] Reeves SA, Chavez-Kappel C, Davis R, Rosenblum M, Israel MA (1992). Developmental regulation of annexin II (Lipocortin 2) in human brain and expression in high grade glioma. Cancer Res.

[11] Sideras K, Bots SJ, Biermann K, Sprengers D, Polak WG, IJzermans JN (2015). Tumour antigen expression in hepatocellular carcinoma in a low-endemic western area. Br J Cancer.

[12] Esposito I, Penzel R, Chaib-Harrireche M, Barcena U, Bergmann F, Riedl S (2006). Tenascin C and annexin II expression in the process of pancreatic carcinogenesis. J Pathol.

[13] Emoto K, Yamada Y, Sawada H, Fujimoto H, Ueno M, Takayama T (2001). Annexin II overexpression correlates with stromal tenascin-C overexpression: a prognostic marker in colorectal carcinoma. Cancer.

[14] Jia JW, Li KL, Wu JX, Guo SL (2013). Clinical significance of annexin II expression in human non-small cell lung cancer. Tumour Biol.

[15] Cole SP, Pinkoski MJ, Bhardwaj G, Deeley RG (1992). Elevated expression of annexin II (lipocortin II, p36) in a multidrug resistant small cell lung cancer cell line. Br J Cancer.

[16] Sharma MR, Koltowski L, Ownbey RT, Tuszynski GP, Sharma MC (2006). Angiogenesis-associated protein annexin II in breast cancer: selective expression in invasive breast cancer and contribution to tumor invasion and progression. Exp Mol Pathol.

[17] Pena-Alonso E, Rodrigo JP, Parra IC, Pedrero JM, Meana MV, Nieto CS (2008). Annexin A2 localizes to the basal epithelial layer and is down-regulated in dysplasia and head and neck squamous cell carcinoma. Cancer Lett.

[18] Rodrigo Tapia JP, Pena Alonso E, Garcia-Pedrero JM, Florentino Fresno M, Suarez Nieto C, Owen Morgan R (2007). Annexin A2 expression in head and neck squamous cell carcinoma. Acta Otorrinolaringol Esp.

[19] Feng JG, Liu Q, Qin X, Geng YH, Zheng ST, Liu T (2012). Clinicopathological pattern and Annexin A2 and Cdc42 status in patients presenting with differentiation and lymphnode metastasis of esophageal squamous cell carcinomas. Mol Biol Rep.

[20] Liu JW, Shen JJ, Tanzillo-Swarts A, Bhatia B, Maldonado CM, Person MD (2003). Annexin II expression is reduced or lost in prostate cancer cells and its re-expression inhibits prostate cancer cell migration. Oncogene.

[21] Mogami T, Yokota N, Asai-Sato M, Yamada R, Koizume S, Sakuma Y (2013). Annexin A4 is involved in proliferation, chemo-resistance and migration and invasion in ovarian clear cell adenocarcinoma cells. PLoS One.

[22] Duncan R, Carpenter B, Main LC, Telfer C, Murray GI (2008). Characterisation and protein expression profiling of annexins in colorectal cancer. Br J Cancer.

[23] Han EK, Tahir SK, Cherian SP, Collins N, Ng SC (2000). Modulation of paclitaxel resistance by annexin IV in human cancer cell lines. Br J Cancer.

[24] Alfonso P, Canamero M, Fernandez-Carbonie F, Nunez A, Casal JI (2008). Proteome analysis of membrane fractions in colorectal carcinomas by using 2D-DIGE saturation labeling. J Proteome Res.

[25] Zimmermann U, Balabanov S, Giebel J, Teller S, Junker H, Schmoll D (2004). Increased expression and altered location of annexin IV in renal clear cell carcinoma: a possible role in tumour dissemination. Cancer Lett.

[26] Kim A, Enomoto T, Serada S, Ueda Y, Takahashi T, Ripley B (2009). Enhanced expression of annexin A4 in clear cell carcinoma of the ovary and its association with chemoresistance to carboplatin. Int J Cancer.

[27] Xin W, Rhodes DR, Ingold C, Chinnaiyan AM, Rubin MA (2003). Dysregulation of the annexin family protein family is associated with prostate cancer progression. Am J Pathol.

[28] Choi CH, Chung JY, Cho H, Kitano H, Chang E, Ylaya K (2015). Prognostic significance of AMP-dependent kinase alpha expression in cervical cancer. Pathobiology.

[29] Choi CH, Chung JY, Park HS, Jun M, Lee YY, Kim BG (2015). Pancreatic adenocarcinoma up-regulated factor expression is associated with disease-specific survival in cervical cancer patients. Hum Pathol.

[30] Lee YY, Kim TJ, Kim JY, Choi CH, Do IG, Song SY (2013). Genetic profiling to predict recurrence of early cervical cancer. Gynecol Oncol.

[31] Ishwaran H, Kogalur UB (2010). Consistency of random survival forests. Stat Probab Lett.

[32] Harrell FE, Lee KL, Mark DB (1996). Multivariable prognostic models: issues in developing models, evaluating assumptions and adequacy, and measuring and reducing errors. Stat Med.

[33] Wang CY, Lin CF (2014). Annexin A2: its molecular regulation and cellular expression in cancer development. Dis Markers.

[34] Mosesson Y, Mills GB, Yarden Y (2008). Derailed endocytosis: an emerging feature of cancer. Nat Rev Cancer.

[35] Jin L, Shen Q, Ding S, Jiang W, Jiang L, Zhu X (2012). Immunohistochemical expression of annexin A2 and S100A proteins in patients with bulky stage IB-IIA cervical cancer treated with neoadjuvant chemotherapy. Gynecol Oncol.

[36] Mohammad HS, Kurokohchi K, Yoneyama H, Tokuda M, Morishita A, Jian G (2008). Annexin A2 expression and phosphorylation are up-regulated in hepatocellular carcinoma. Int J Oncol.

[37] Vishwanatha JK, Chiang Y, Kumble KD, Hollingsworth MA, Pour PM (1993). Enhanced expression of annexin II in human pancreatic carcinoma cells and primary pancreatic cancers. Carcinogenesis.

[38] Zhao P, Zhang W, Tang J, Ma XK, Dai JY, Li Y (2010). Annexin II promotes invasion and migration of human hepatocellular carcinoma cells in vitro via its interaction with HAb18G/CD147. Cancer Sci.

[39] Shiozawa Y, Havens AM, Jung Y, Ziegler AM, Pedersen EA, Wang J (2008). Annexin II/annexin II receptor axis regulates adhesion, migration, homing, and growth of prostate cancer. J Cell Biochem.

[40] Lokman NA, Elder AS, Ween MP, Pyragius CE, Hoffmann P, Oehler MK (2013). Annexin A2 is regulated by ovarian cancer-peritoneal cell interactions and promotes metastasis. Oncotarget.

[41] Mai J, Waisman DM, Sloane BF (2000). Cell surface complex of cathepsin B/annexin II tetramer in malignant progression. Biochim Biophys Acta.

[42] Oliferenko S, Paiha K, Harder T, Gerke V, Schwarzler C, Schwarz H (1999). Analysis of CD44-containing lipid rafts: recruitment of annexin II and stabilization by the actin cytoskeleton. J Cell Biol.

[43] Walboomers JM, Jacobs MV, Manos MM, Bosch FX, Kummer JA, Shah KV (1999). Human papillomavirus is a necessary cause of invasive cervical cancer worldwide. J Pathol.

[44] Raff AB, Woodham AW, Raff LM, Skeate JG, Yan L, Da Silva DM (2013). The evolving field of human papillomavirus receptor research: a review of binding and entry. J Virol.

[45] Dziduszko A, Ozbun MA (2013). Annexin A2 and S100A10 regulate human papillomavirus type 16 entry and intracellular trafficking in human keratinocytes. J Virol.

[46] Woodham AW, Taylor JR, Jimenez AI, Skeate JG, Schmidt T, Brand HE (2015). Small molecule inhibitors of the annexin A2 heterotetramer prevent human papillomavirus type 16 infection. J Antimicrob Chemother.

[47] Hill WG, Kaetzel MA, Kishore BK, Dedman JR, Zeidel ML (2003). Annexin A4 reduces water and proton permeability of model membranes but does not alter aquaporin 2-mediated water transport in isolated endosomes. J Gen Physiol.

[48] Sohma H, Creutz CE, Gasa S, Ohkawa H, Akino T, Kuroki Y (2001). Differential lipid specificities of the repeated domains of annexin IV. Biochim Biophys Acta.

[49] Piljic A, Schultz C (2006). Annexin A4 self-association modulates general membrane protein mobility in living cells. Mol Biol Cell.

[50] Kaetzel MA, Chan HC, Dubinsky WP, Dedman JR, Nelson DJ (1994). A role for annexin IV in epithelial cell function. Inhibition of calcium-activated chloride conductance. J Biol Chem.

[51] Jeon YJ, Kim DH, Jung H, Chung SJ, Chi SW, Cho S (2010). Annexin A4 interacts with the NF-kappaB p50 subunit and modulates NF-kappaB transcriptional activity in a Ca2 + −dependent manner. Cell Mol Life Sci.

[52] Choi CH, Sung CO, Kim HJ, Lee YY, Song SY, Song T (2013). Overexpression of annexin A4 is associated with chemoresistance in papillary serous adenocarcinoma of the ovary. Hum Pathol.

