# Supplementary material for: Prognostic significance of annexin A2 and annexin A4 expression in patients with cervical cancer
Source: BMC Cancer. 2016 Jul 11;16:448. doi: 10.1186/s12885-016-2459-y (PMC4940752; doi:10.1186/s12885-016-2459-y)
Supplement: Additional file 1: Figure S1. — Histoscore distribution of annexin A2 (ANXA2) and annexin A4 (ANXA4) expression using a quantitative image analysis. Dashed vertical lines indicate the chosen cut-off values (ANXA2 = 94 and ANXA4 = 51). Figure S2. Kaplan-Meier survival curves according to annexin A2 (ANXA2) and annexin A4 (ANXA4) mRNA expression. Data were retrieved from the GEO (http://www.ncbi.nlm.nih.gov/geo/query/acc.cgi?acc=GSE44001) and TCGA (RNA-seq databases (version: 2015-02-24). The mRNA expression values were dichotomized according to quartile values (lower than 25 percentile vs. higher than 75 percentile). Figure S3. Correlations between annexin A2 (ANXA2) and annexin A4 (ANXA4) mRNA and protein expression. Positive correlations were noted in both ANXA2 (Spearman’s rho = 0.273, p < 0.001) and ANXA4 (Spearman’s rho = 0.293, p < 0.001). A subgroup analysis showed a positive correlation between ANXA2 expression and squamous cell carcinoma (Spearman’s rho = 0.255, p < 0.001) and ANXA4 expression and adenocarcinoma (Spearman’s rho = 0.389, p = 0.002). Figure S4. ANXA2 and ANXA4 mRNA expression according to cancer types. (Data from cBioPortal Cancer Genomics: www.cbioportal.org). Figure S5. ANXA2 and ANXA4 mRNA expression according to HPV type infected. Data was retrieved from TCGA (RNA-seq database version 2015-02-24). (PPTX 1250 kb) [file 12885_2016_2459_MOESM1_ESM.pptx]

## Slide 1
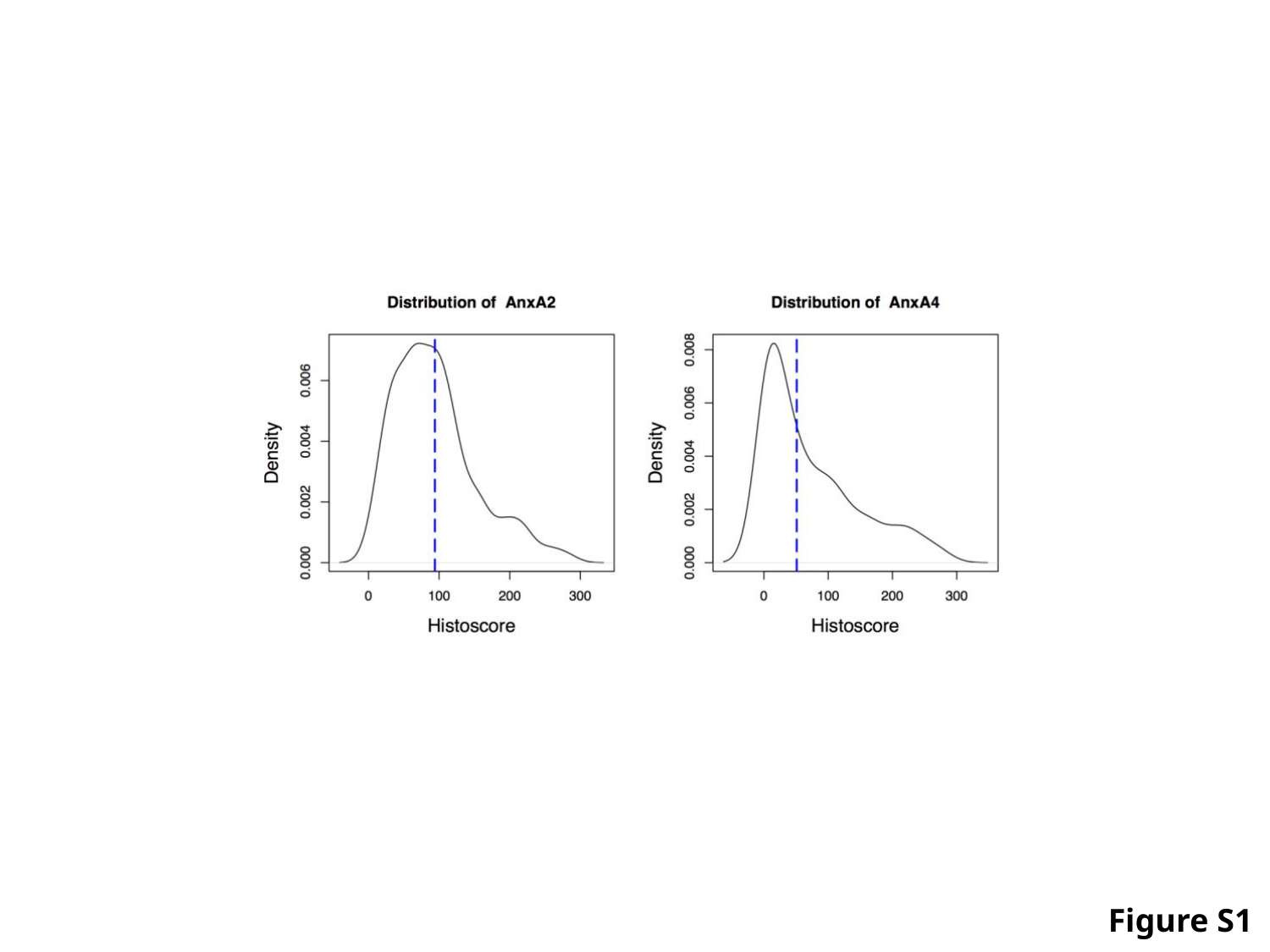

Figure S1

## Slide 2
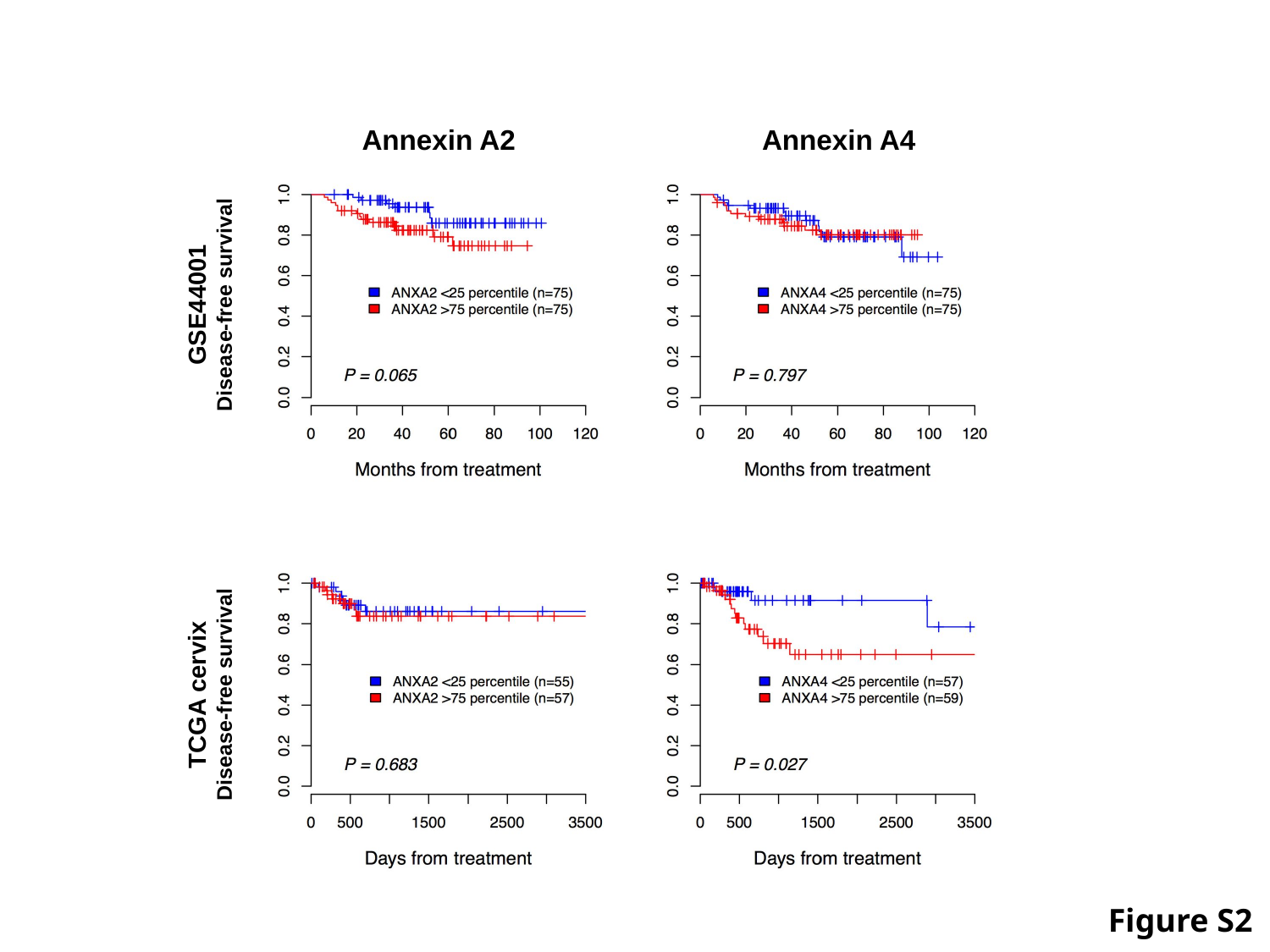

Annexin A2
Annexin A4
GSE44001
Disease-free survival
TCGA cervix
Disease-free survival
Figure S2

## Slide 3
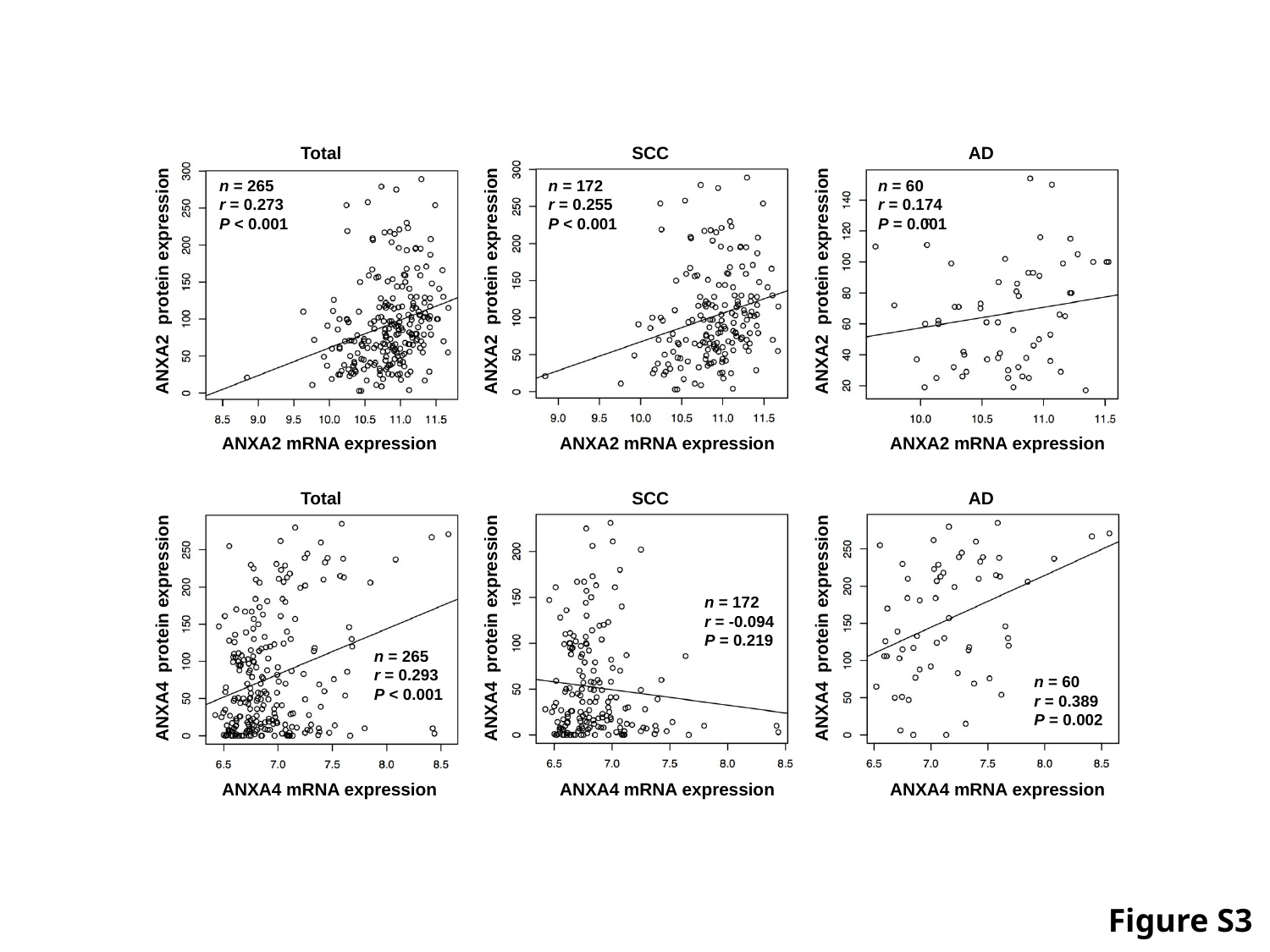

Total
SCC
AD
n = 265
r = 0.273
P < 0.001
n = 172
r = 0.255
P < 0.001
n = 60
r = 0.174
P = 0.001
ANXA2 protein expression
ANXA2 protein expression
ANXA2 protein expression
ANXA2 mRNA expression
ANXA2 mRNA expression
ANXA2 mRNA expression
Total
SCC
AD
n = 172
r = -0.094
P = 0.219
ANXA4 protein expression
ANXA4 protein expression
ANXA4 protein expression
n = 265
r = 0.293
P < 0.001
n = 60
r = 0.389
P = 0.002
ANXA4 mRNA expression
ANXA4 mRNA expression
ANXA4 mRNA expression
Figure S3

## Slide 4
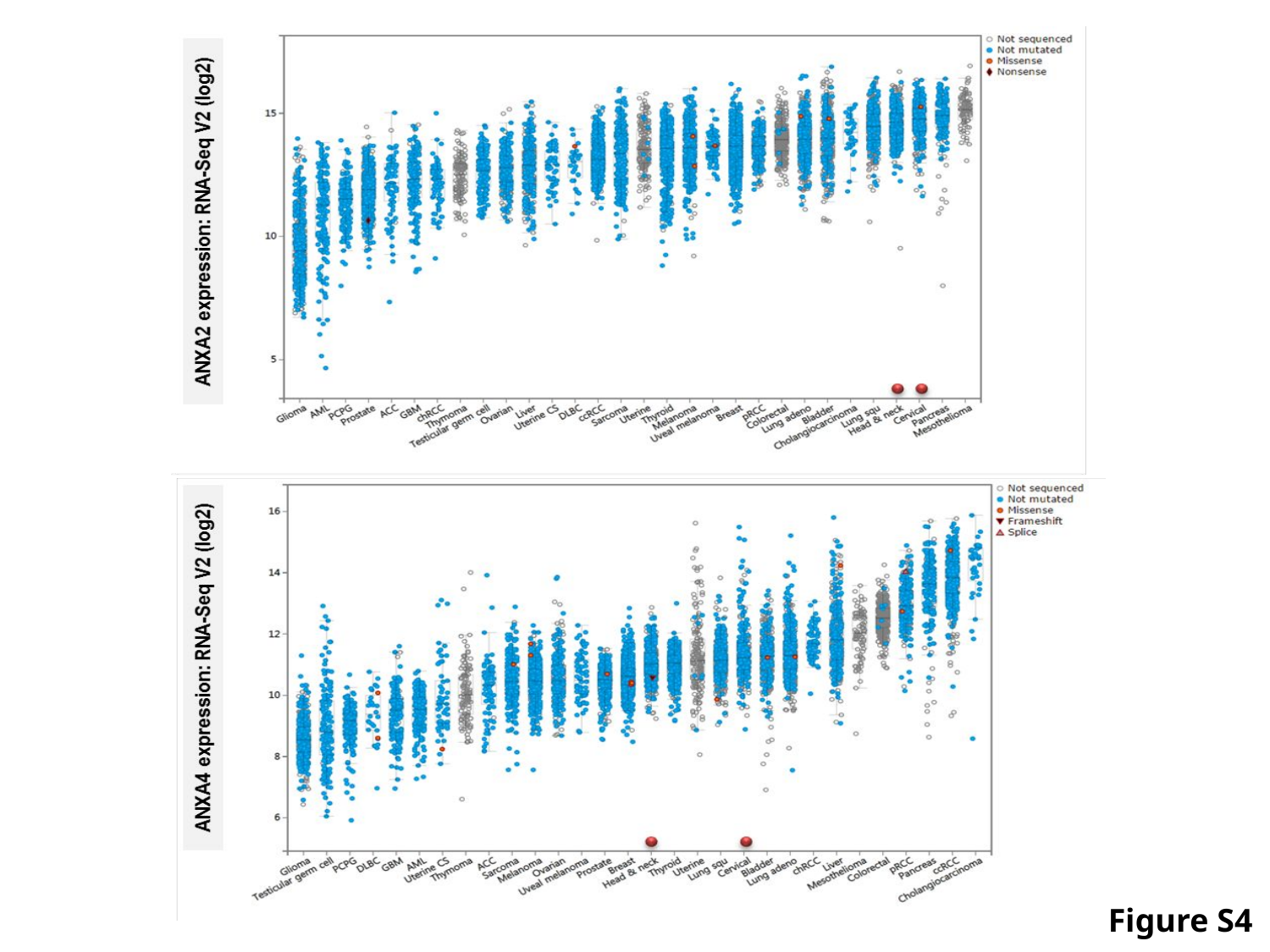

Figure S4

## Slide 5
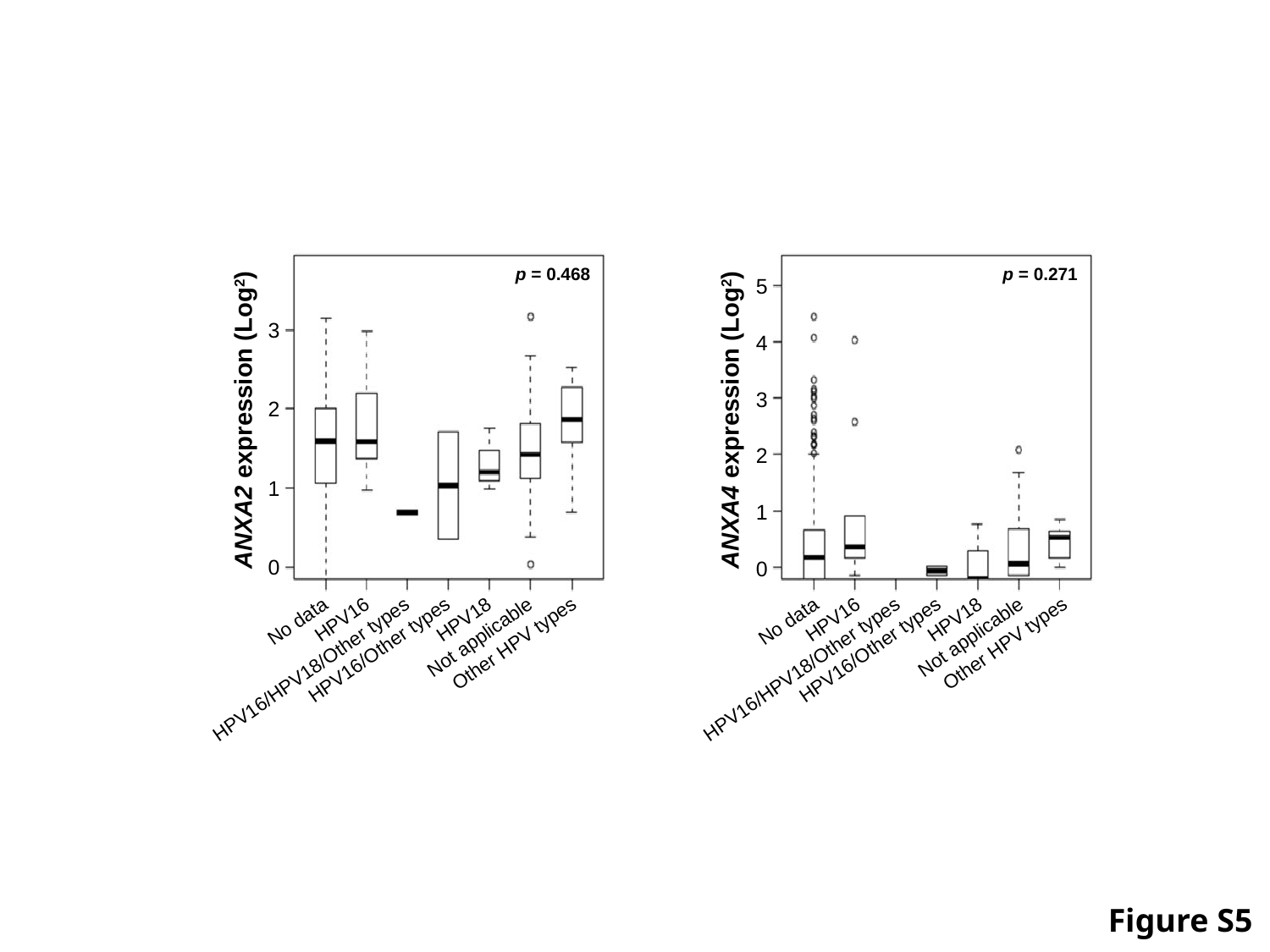

p = 0.468
p = 0.271
5
3
4
3
2
ANXA2 expression (Log2)
ANXA4 expression (Log2)
2
1
1
0
0
HPV16
HPV18
No data
Not applicable
Other HPV types
HPV16/Other types
HPV16/HPV18/Other types
HPV16
HPV18
No data
Not applicable
Other HPV types
HPV16/Other types
HPV16/HPV18/Other types
Figure S5
