# Supplementary material for: Prognostic significance of annexin A2 and annexin A4 expression in patients with cervical cancer
Source: BMC Cancer. 2016 Jul 11;16:448. doi: 10.1186/s12885-016-2459-y (PMC4940752; doi:10.1186/s12885-016-2459-y)
Supplement: Additional file 2: Table S1. — Univariate and multivariate analyses of the association between prognostic variables and overall survival in patients with cervical cancer. (DOCX 15 kb) [file 12885_2016_2459_MOESM2_ESM.docx]

**Table S1** Univariate and multivariate analyses of the association between prognostic variables and overall survival in patients with cervical cancer

| Risk factor | Univariate |  | Multivariate | |
| --- | --- | --- | --- | --- |
|  | Hazard ratio [95%CI] | *p* value | Hazard ratio [95%CI] | *p* value |
| FIGO stage (> IIB) | 2.49 [0.9 - 6.87] | 0.078 | 1.66 [0.56 - 4.92] | 0.364 |
| Cell type (AD) | 4.52 [1.85 - 11.06] | 0.001 | 6.68 [2.58 - 17.32] | <0.001 |
| LN metastasis | 2.93 [1.22 - 7.06] | 0.017 | 2.65 [1.02 - 6.88] | 0.045 |
| Tumor size (> 4 cm) | 1.48 [0.57 - 3.86] | 0.419 | 0.91 [0.32 - 2.56] | 0.852 |
| PM involvement | 2.83 [0.94 - 8.48] | 0.064 | 1.85 [0.57 - 6.08] | 0.309 |
| ANXA2+ | 1.68 [0.69 - 4.06] | 0.250 | 2.09 [0.77 - 5.67] | 0.146 |
| ANXA4+ | 2.73 [1.05 - 7.1] | 0.040 | 1.4 [0.44 - 4.46] | 0.566 |
| ANXA2+/ANXA4+ | 4.37 [1.16 - 16.52] | 0.030 | 2.15 [0.5 - 9.34] | 0.305 |

*CI* confidential interval, *ANX* annexin, *FIGO* International Federation of Gynecology and Obstetrics, *AD* adenocarcinoma, *LN* lymph node, *PM* parametrial
